# Effect of Saturated Tea Waste and Perlite Particles on Early Age Hydration of High-Strength Cement Mortars

**DOI:** 10.3390/ma12142269

**Published:** 2019-07-15

**Authors:** Sadam Hussain Jakhrani, Hong Gi Kim, In Kyu Jeon, Jae Suk Ryou

**Affiliations:** Department of Civil and Environmental Engineering, Hanyang University, Seoul 04763, Korea

**Keywords:** black tea waste, hydration, setting times, absorption, strength, ultrasonic pulse velocity

## Abstract

The main purpose of this work is to study the effect of saturated black tea waste and perlite on controlling the rapid heat of hydration in high-strength cement mortars at early ages. Tea waste and perlite were investigated as internal curing agents in different mixes. Mortar specimens with two different sizes of tea waste and perlite particles with 1 and 3% by volume of cement were added in different mixes to find their effect on early age hydration. The rising interior temperature, setting times, and strength parameters were evaluated. Results showed that the mix specimens that contained 3% tea waste and perlite particles significantly delayed the hydration process by minimizing internal temperature and extended setting times of different specimens. However, their usage had a slightly adverse impact on compressive and flexural strengths. It was observed that the specimens made with coarser particles of tea waste and perlite were more helpful to control early age rapid hydration than the specimens made with finer particles, whereas the specimens made with finer particles had slightly higher strengths than the specimens made with coarser particles. Hence, the coarser particles are recommended to be used in high-strength mortars to mitigate the early age rapid heat of hydration.

## 1. Introduction

High-strength mortars (HSMs) and high-strength concretes (HSCs) are widely-used cement composites in the modern construction industry. HSMs and HSCs, however, are susceptible to micro cracking due to rapid heat release rates during the hydration process at early ages [1]. According to Pacheco et al. [2] and Tran et al. [3], the major weaknesses of HSMs and HSCs are their extraordinary brittleness, low tensile strength, and high cracking, which are caused by early age shrinkage due to rapid hydration, all of which limit their use in mega-construction projects. To overcome early age shrinkages due to rapid hydration, saturated lightweight aggregates (LWAs), superabsorbent polymers (SAPs), and superfine powders (SFPs) are commonly used, and they act as internal curing agents (ICAs). Mass concrete structures face thermal shrinkage because of temperature differences between the surface of the concrete and the interior of the concrete, mainly due to dissipation of the heat of hydration [4]. The basic purpose of ICAs in HSMs and HSCs is to release stored water during the hydration process to delay rapid hydration and minimize leading interior temperatures [5,6,7,8]. Other than commonly-used ICAs, Moon et al. [9] added slag with other supplementary cementitious materials (SCMs) to concrete to reduce the early heat release rates. Xu et al. [10] studied the effects of ground granulated blast-furnace slag (GGBS) and fly ash (FA) on the early hydration and mechanical strength of concrete. Miao et al. [11] used different types of SCMs and ZY-type™ expansive agent in their study to determine their effects on early hydration kinetics. They found that the ZY-type™ expansive agent released more heat than a control mix and other mixes that contained SCMs. The basic purpose of different ICAs is to store extra water and steadily release that water during hydration [12]. The addition of ICAs enables a higher ultimate degree of hydration to be obtained at later ages and, finally, facilitates the formation of air voids and pores in concrete to resist frost action [13,14]. The use of lignocellulosic materials as fillers or reinforcements in polymers or ceramic matrices is encouraged, because they have some advantages, like being lightweight, low cost, easily recyclable, having low thermal expansion, being non-abrasive to machinery, and having non-brittle fracture [15].

The literature shows that the pre-saturated porous lightweight materials can be used to control rapid hydration in the form of internal curing agents. In this study, tea waste is used for internal curing purposes, because it has similar properties as other internal curing agents have. According to a report made by Food and Agriculture Organization Corporate Statistical Database (FAOSTAT) [16], tea is the second most commonly-consumed beverage globally, after water. Global tea production increases annually. It increased by 4.4% annually over the decade following 2007, reaching 5.77 million metric tons in 2016. China was the leading tea producer, producing 42% of tea worldwide. Moreover, Chinese production over the last decade (2007–2016) nearly doubled (1.17 million metric tons to 2.44 million metric tons), which ultimately generated a huge amount of waste, and that waste is a burden on the environment. Moreover, numerous researchers used tea waste in their studies, found appropriate outcomes, and proposed recommendations. The composition of tea waste leaves was studied by Duke and Atchely [17]. Adi and Noor [18] recognized that tea and coffee wastes are disposed of in compost or in landfill disposal, which is a helpful environment for vermicomposting to become more stable. They recommended the proper usage of tea and coffee waste. It was found that fresh tea leaves contain about 27% crude fiber, 22% polyphenols, 17% protein, 6% pectin, and 5.6% ash per 100 gram. Demir [19] investigated the effects of processed waste tea (PWT) addition in building bricks. It was found that the addition of 5% PWT was more helpful in enhancing the strength properties of bricks in unfired and fired condition, while, the porosity and bulk densities were found to be increased and decreased, respectively. They established that the addition of organic PWT did not show any harmful effects on other parameters of bricks. Moreover, Huang et al. [20] used black tea waste powder (BTWP) in hemihydrate gypsums. They investigated the properties of black tea waste powder and found that BTWP contains 59.2% carbohydrates, 26.7% proteins, and 18% organic acids, by mass. It was established that the addition of a high amount of BTWP increases setting times and densities and decreases the compressive and flexural strengths of specimens at later-ages. 

This study uses black tea waste in an effort to minimize its adverse impacts on the environment as suggested by Adi and Noor [18]. The porous nature and high absorption capability of tea waste make it a suitable material to be used as an internal curing agent in high-strength mortars to control early age rapid hydration and the leading interior high temperatures. Moreover, perlite is a commonly-used lightweight aggregate in concrete. Its usage along with other lightweight aggregates in normal concretes, self-compacting concretes, and in ultra-high performance concretes was proven in many previous studies [21,22,23,24,25]. In this study, it is used for only comparison purposes with tea waste particles.

## 2. Experimental Work

### 2.1. Materials and Methods

**Figure 1:**
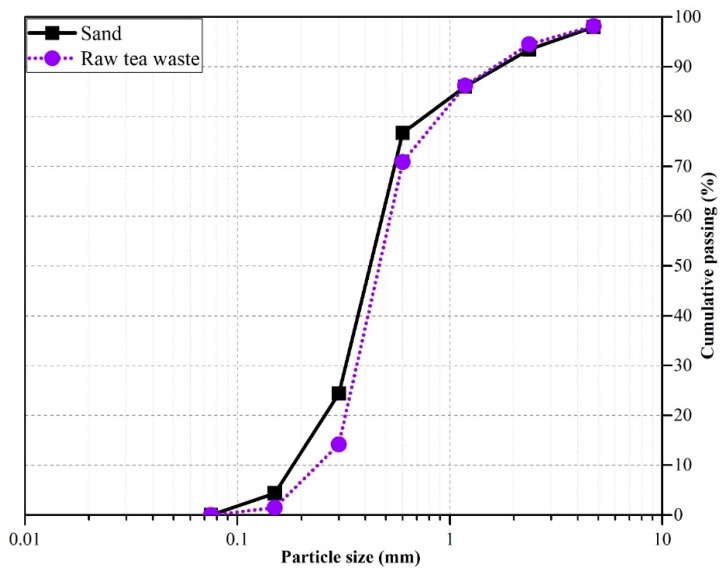
Gradation curves of sand and raw tea waste.

Ordinary Portland cement (OPC) meeting the requirements of ASTM C150 [26] was used as the major binder, and natural river sand was used as the fine aggregate. The particles of natural river sand were passed through ASTM Sieve No. 4 and retained on ASTM Sieve No. 200. Black tea waste (obtained from domestic waste in local areas of Seoul) and perlite (obtained from the local market) particles were used as internal curing agents to store water inside the particles and minimize interior temperatures during hydration at early ages. Liquid-type superplasticizer (S.P) was used as a high-range water-reducing admixture. Mixes made with tea waste and perlite particles were labeled as TW and P, respectively. Two different size ranges were selected for both types of particles. Tea waste particles were labeled TW30 and TW50, respectively, whereas perlite particles were labeled P30 and P50, respectively. The number 30 indicates that the particles were passed through ASTM Sieve No. 4 and were retained on Sieve No. 30, whereas the number 50 indicates that the particles were passed through ASTM Sieve No. 30 and were retained on Sieve No. 50. The amount of tea waste and perlite particles added into mixes was 1% and 3% by volume of cement. The obtained physical properties of the sand, tea waste, and perlite are provided in Table 1, and gradation curves for sand and raw tea waste are shown in Figure 1.

### 2.2. Mix Proportions

Nine mixes were prepared for this work. The control mix contained ordinary Portland cement as the only binder and natural river sand as the fine aggregate. Mixes TW30-1, TW30-3, TW50-1, and TW50-3 contained cement as the binder, sand as the fine aggregate, and tea waste (TW30 and TW50) as the internal curing agent. The same amount of materials was used in the mixes P30-1, TW30-3, P50-1, and TW50-3 that contained perlite (P30 and P50) as the internal curing agent. Tea waste and perlite particles were added up to 3% by volume of cement. The total water/cement ratio (w/c) = 0.20 was adjusted as the mixing water; however, some part of the mixing water was used as internal curing water to saturate ICA particles fully before mixing. Detailed mix proportions are given in Table 2.

### 2.3. Mixing and Molding

Mixing was performed manually in the laboratory at room temperature (25 ± 2°C). As discussed previously, nine mix specimens were made for this study. For the control mix, ingredients (OPC and saturated surface dry (SSD) sand) were mixed for one minute. After that, the required amounts of water and superplasticizer were added and mixed for an additional two minutes. However, the mixing technique used for the remaining eight mix specimens (TW30-1, TW30-3, TW50-1, TW50-3, P30-1, P30-3, P50-1, and P50-3) was slightly different. For the mix specimens that contained tea waste (TW30 and TW50) particles, the particles were fully saturated in water for one hour before use. Then, saturated tea waste particles, SSD sand, and cement were mixed together in water for two minutes. Finally, water and S.P were added, and mixing was done for an additional two minutes. After mixing for a total of five minutes, the fresh mortar was taken out and placed in molds. A similar procedure was carried out for the mixes that contained perlite (P30 and P50) particles. Three (03) replicate specimens were tested for each mix design, and their averages values are reported in the Results and Discussion Sections.

### 2.4. Tests of Fresh and Hardened Mortars

To measure the heat release rates, fresh mortar mixes were placed in small plastic bottles with a height of 50 mm and a diameter of 30 mm. Before putting the mortar into bottles, bottles were wrapped in an insulating sheet. Early heat release rate or temperature rise was measured for up to 40 h by a semi-adiabatic method [9]. ASTM C191 [27] is a standard test method for measuring setting times of pastes, but in this study, we used this method to measure that for mortars. Oven dry (OD) densities, saturated surface dry (SSD) densities, and weights and absorption capacity of specimens were measured on prismatic mortar specimens. After 24 h of casting, the specimens were demolded and placed in water for 24 h. They were taken out from the water after 24 h, then their surface was dried with a dry towel and their weight measured. After that, specimens were put in a drying oven for a further 24 h at a temperature of 105 °C. The next day, specimens were taken out from the drying oven and weighed. In this way, oven dry (OD) and saturated surface dry (SSD) densities and weights were obtained. By calculating the difference between two weights, the absorption percentage was calculated by using Equation (1) given in Section 3.4. Cubic specimens of 50 × 50 × 50 mm^3^ were made. The compressive strengths of specimens after demolding and curing in water for 3 days were measured by the method stated in ASTM C109 [28]. Prismatic mortar specimens of 40 × 40 × 160 mm^3^ were used in flexural strength and ultrasonic pulse velocity (UPV) tests. The methods used to test flexural strength and UPV conformed to ASTM C348 [29] and ASTM C517 [30], respectively. 

## 3. Results and Discussion

### 3.1. Interior Temperature and Hydration 

**Figure 2:**
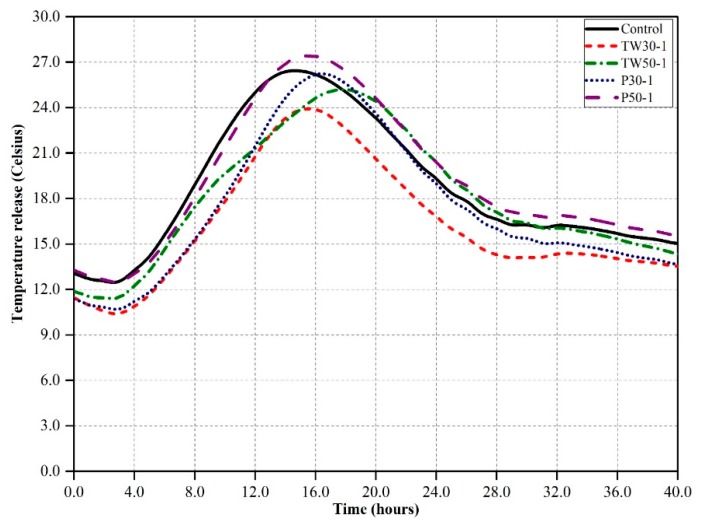
Temperature release of the control mix and mixes containing 1% internal curing agents (ICA) over 40 h.

**Figure 3:**
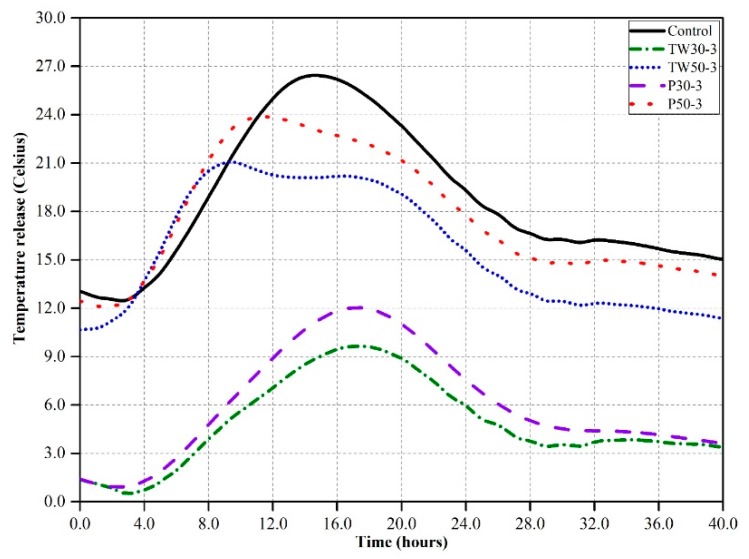
Temperature release of the control mix and mixes containing 3% ICA over 40 h.

The interior temperature release rate and hydration rate of cement mortars with and without tea waste and perlite particles were obtained using the semi-adiabatic calorimetry method. The results are shown in Figure 2 and Figure 3. Figure 2 shows the heat release rates over 40 h of five mixes (control, TW30-1, TW50-1, P30-1, and P50-1) that contained 1% by volume of cement with coarser and finer particles of tea waste and perlite. The maximum temperature in the control mix was around 26.0 °C within 15.0 h of casting. The other four mixes showed similar behavior to the control mix, but the maximum temperature peaks were slightly lower than the control mix, except for P50-1, which had a maximum temperature peak around 27.0 °C within 15.5 h. The mixes TW30-1, TW50-1, and P30-1 had maximum temperatures of around 24.0 °C, 25.0 °C, and 26.5 °C after 15.0, 18.0, and 16.0 h, respectively.

Figure 3 shows the heat release rates of five mixes (control, TW30-3, TW50-3, P30-3, and P50-3) that contained 3% tea waste (TW30 and TW50) and perlite (P30 and P50) particles by volume of cement over 40 h. Mixes TW30-3 and P30-3, which contained coarser particles of tea waste and perlite had very low temperatures of around 9 °C and 12 °C at 17 h, respectively. However, the temperature values of the other two mixes (TW50-3 and P50-3), which contained finer particles, were higher than those of the two mixes that contained coarser particles. The temperature values of TW50-3 and P50-3 were 21 °C and 24 °C at 10 h, respectively. 

Numerous authors have tried to capture the real processes that occur during hydration. Zou et al. [31] recommended that the hydration of cement can be slowed down by internal curing at early ages, but with the passage of time, the hydration process increases its rate due to the release of water from internal curing agents. Xu et al. [10] found that the addition of GGBS accelerated the hydration process and attributed this to a dilution and filler effect; however, the addition of 55% and 70% FA slowed down the hydration process, which was attributed to the sensitivity of FA to inner temperatures. Oh and Choi [32] used SAPs as an internal curing agent in alkali-activated slag mortars. They found no significant effect of SAPs on hydration at early ages, and they recommended that it was due to swallowing of internal curing water by SAPs and that water did not contribute to the hydration process. We concluded from the results that the addition of 3% coarser tea waste and perlite particles reduced the temperature peaks by up to 10.0 °C and delayed the early hydration process by up to 2.0 h; however, finer particles did not contribute much to the reduction of the inner temperature, nor did they delay hydration. Moreover, tea waste particles were found to be more suitable for mitigating rapid hydration and minimizing inner temperatures than perlite particles at early ages. The delay in hydration and reduction in interior temperature was attributed to the release of internally-cured water from the pores of the tea waste at a time when the hydration process needed further water for complete and proper hydration.

### 3.2. Setting Times

**Figure 4:**
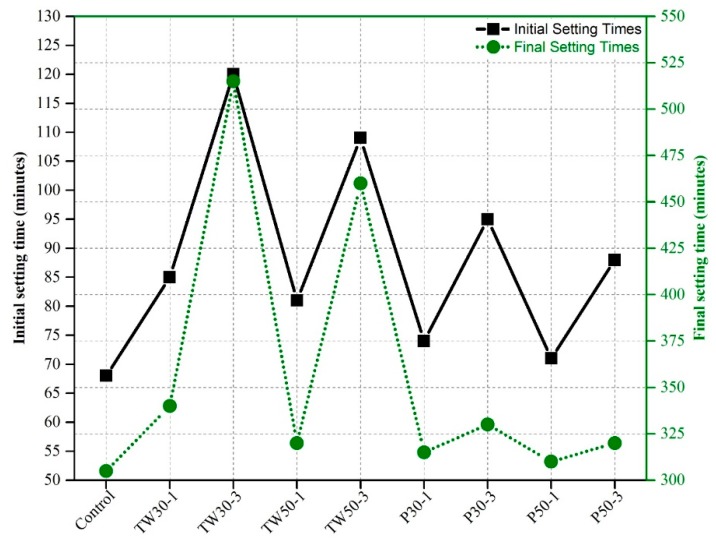
Initial and final setting times of specimens.

The setting times of all nine mixes (control, TW30-1, TW30-3, TW50-1, TW50-3, P30-1, P30-3, P50-1, and P50-3) are shown in Figure 4. The initial and final setting times of the control mix were 68 and 305 min, respectively. The initial and final setting times of the remaining eight mixes were 103 and 375 min, 215 and 940 min, 91 and 350 min, 200 and 460 min, 84 and 335 min, 115 and 401 min, 79 and 328 min, and 103 and 388 min, respectively.

Wyrzykowski et al. [33] used bottom ash as an internal curing material. The final setting time of the mix that contained bottom ash with a 0.35 w/c ratio and 2.3% of superplasticizer was 690 min as compared to the control mix, which was 420 min with the same w/c ratio and 0.5% superplasticizer. SAPs have been used in various mixes with different ingredients. The addition of an increasing amount of SAP has been shown to enhance the initial and final setting times as compared to control mixes [32,34,35]. Huang et al. [20] used black tea waste in hemihydrate gypsums and found that utilization of black tea waste powder increased setting times. Liu et al. [36] used cenospheres as an internal curing agent. They found that 9% cenospheres slightly increased initial and final setting times as compared to control specimens. Darweesh et al. [37] used different percentages of perlite powder in cement pastes and reported longer initial and final setting times. Erdem et al. [23] used perlite as a pozzolanic addition with different amounts in cement pastes and reported longer setting times of cement pastes. 

Our results indicated that the addition of saturated tea waste and perlite particles increased both the initial and final setting times of specimens. The setting time of the control mix was the lowest among all mixes, whereas the TW30-3 mix had the highest setting time. The integration of 3% pre-saturated coarser ICA (tea waste and perlite) particles resulted in longer setting times than the addition of 1% ICA. The addition of pre-saturated particles filled all pores of the ICAs with water, which caused a delay in setting time. Coarser tea waste particles were more helpful in extending setting times than finer tea waste particles. 

### 3.3. Densities of Specimens

**Figure 5:**
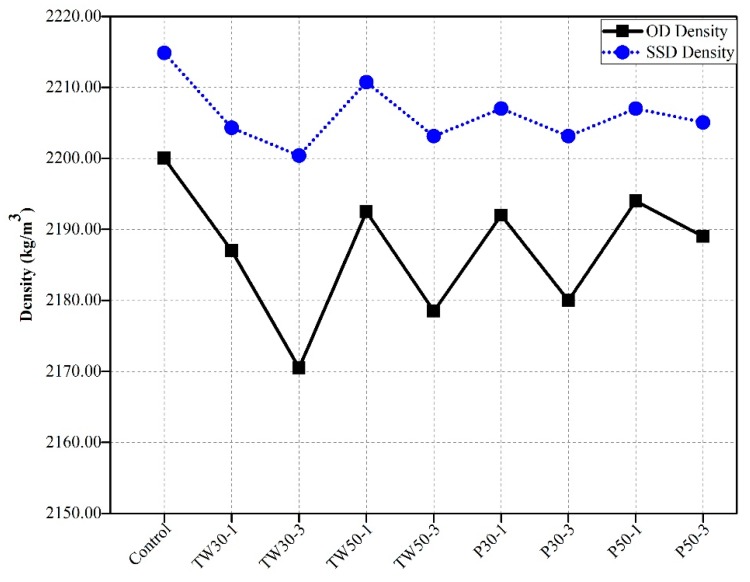
Oven dry and saturated surface dry densities of specimens.

Figure 5 shows the OD and SSD densities of prismatic mortar specimens of all nine mixes. The OD and SSD densities of the control mix were 2200 kg/m^3^ and 2214.84 kg/m^3^, respectively. The OD and SSD densities of the remaining eight mixes (control, TW30-1, TW30-3, TW50-1, TW50-3, P30-1, P30-3, P50-1, and P50-3) were 2187.00 kg/m^3^ and 2204.30 kg/m^3^, 2170.50 kg/m^3^ and 2200.39 kg/m^3^, 2192.50 kg/m^3^ and 2210.79 kg/m^3^, 2178.50 kg/m^3^ and 2203.12 kg/m^3^, 2192.00 kg/m^3^ and 2207.07 kg/m^3^, 2180.00 kg/m^3^ and 2203.12 kg/m^3^, 2194.00 kg/m^3^ and 2207.03 kg/m^3^, and 2189.00 kg/m^3^ and 2205.08 kg/m^3^, respectively. 

Al Saffar et al. [8] and Mechtcherine et al. [38] recognized that the densities of high-performance concretes containing internal curing agents decrease at early ages, and they established that this reduction in densities might be due to the low densities of internal curing agents. However, some studies showed an increase in densities at later-ages and attributed this to the formation of a very dense microstructure at later stages. Agostini et al. [39] used treated sediment aggregate (TSA) as a curing agent and found that usage of a high amount of TSA reduced the density of concrete and increased its porosity. They concluded that internal water curing increases the density of specimens because continuous water supply enhances hydration and results in the formation of a denser microstructure at later ages. Gesog˘lu et al. [40] replaced normal weight fine aggregates with lightweight fine aggregates from 0%–100% by mass in concrete. Reduction in the unit weight of specimens was reported when the amount of lightweight fine aggregate in concrete increased and obtained the unit weight of specimens of 1773 kg/m^3^ with 100% replacement and 2035 kg/m^3^ with 0% replacement.

In our study, a similar trend was observed with respect to the reviewed literature. The control mix had higher densities under OD and SSD conditions as compared to the other eight mixes. This proved that the addition of lightweight aggregates reduced the densities of specimens. Hence, the higher densities of control mix specimens at early ages were attributed to the high densities of the materials in the mixes and early hydration, leading to the formation of denser micro phase structures. Moreover, the densities of specimens containing tea waste were lower than those containing perlite particles, which was also attributed to differences in the densities of each type of these materials. 

### 3.4. Unit Weight and Absorption

**Figure 6:**
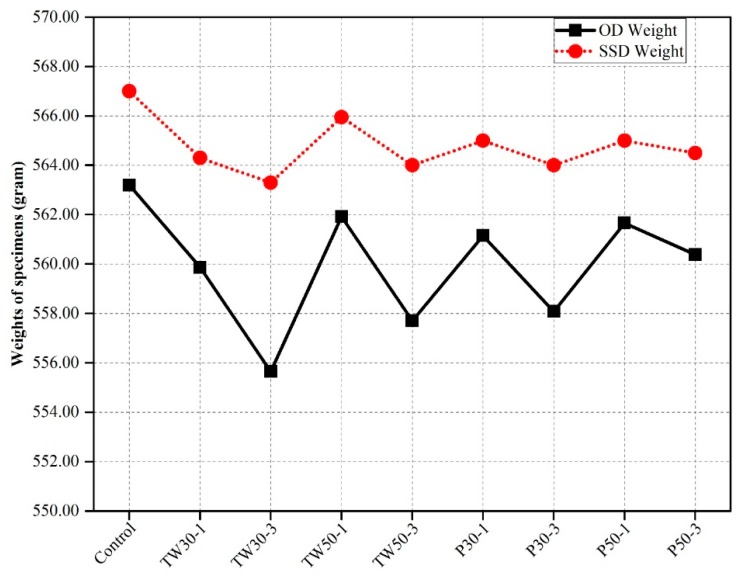
Oven dry (OD) and saturated surface dry (SSD) unit weights of specimens.

**Figure 7:**
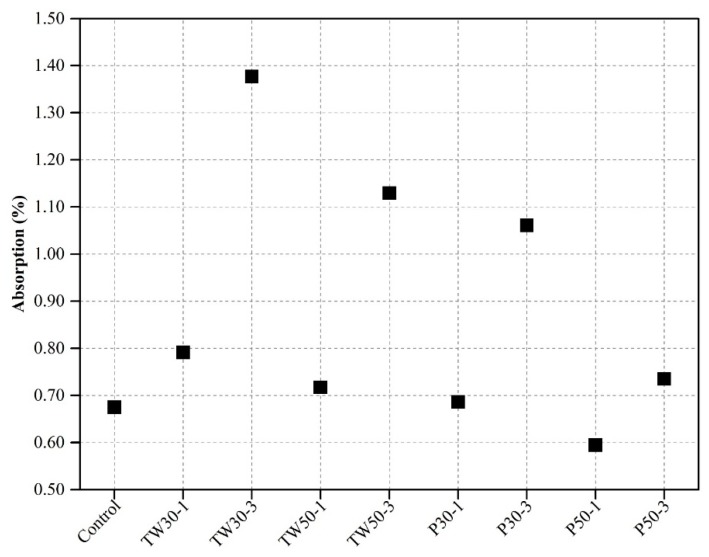
Absorption ability of specimens.

The weight and water absorption rate of prismatic mortar specimens are shown in Figure 6 and Figure 7. Figure 6 shows the average weights of all nine mixes (control, TW30-1, TW30-3, TW50-1, TW50-3, P30-1, P30-3, P50-1, and P50-3) under OD and SSD conditions. The average OD weights (W_OD_) and SSD weights (W_SSD_) of prismatic mortar specimens of the control mix were 563.20 g and 567.00 g, respectively. The average OD and SSD weights of the remaining eight mixes were 559.87 g and 564.30, 555.65 g and 563.30 g, 561.92 g and 565.95 g, 557.70 g and 564.0 g, 561.15 g and 565.00 g, 558.08 g and 564.00 g, 561.66 g and 565.00 g, and 560.38 g and 564.50 g, respectively.

From the weight outcomes, we concluded that the control mix had higher weight values than the other mixes. This could be due to the presence of heavy-weight ingredient materials and long-term or greater compaction during placement of the mortar in molds, as was found in the previous section, which was attributed to the high densities of specimens and ingredients. The specimens made with tea waste and perlite particles were slightly lighter than the control mix, which might be due to the porous nature of the internal curing agents (tea waste and perlite particles) or low compaction at the time of placement in the mold.

Figure 7 shows the absorption percentage (%) of mix specimens. The absorption rate of mix specimens was obtained using Equation (1) below:

(1) $$Absorption\;\left(\%\right)=\left(\frac{W_{SSD}-W_{OD}}{W_{OD}}\right)\times 100$$

W_SSD_ and W_OD_ values used in this equation were obtained from the OD and SSD weights of specimens used for weight measurements.

The absorption (%) of the control mix was observed to be 0.67%. The absorption values of the remaining eight mixes (TW30-1, TW30-3, TW50-1, TW50-3, P30-1, P30-3, P50-1, and P50-3) were 0.79%, 1.38%, 0.72%, 1.14%, 0.68% 1.07%, 0.59%, and 0.74%, respectively.

Rashad [22] concluded that the inclusion of expanded perlite in the matrix increased the absorption capabilities and porosity of matrix, which resulted in a reduction of mechanical strength.

From the absorption results, we concluded that the control mix had a lower absorption rate than most other mixes. This was attributed to its greater density. However, the higher absorption rate of other mixes was attributed to the high absorption rate of the ingredients used. Specimens made with coarser tea waste had a higher absorption than specimens made with perlite particles. This was due to the high absorption rate of coarser particles than finer particles due to the large pore sizes.

### 3.5. Compressive Strength

**Figure 8:**
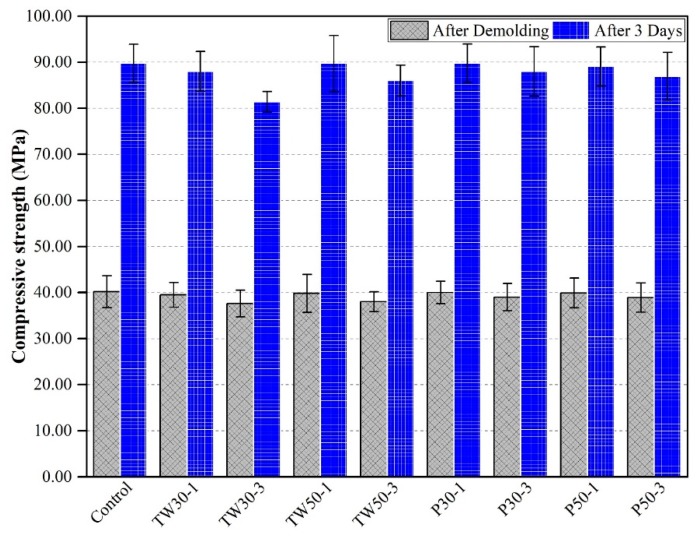
Compressive strength of specimens.

The average compressive strengths of all mix specimens after demolding and curing in water for three days are shown in Figure 8. The compressive strengths of the control mix after demolding and curing in water for three days were 40.20 MPa and 89.76 MPa, respectively. The compressive strengths of the remaining eight mixes (TW30-1, TW30-3, TW50-1, TW50-3, P30-1, P30-3, P50-1, and P50-3) were 39.50 MPa and 88.00 MPa, 37.60 MPa and 81.40 MPa, 39.80 MPa and 89.70 MPa, 38.00 MPa and 86.00 MPa, 40.00 and 89.74 MPa, 39.00 MPa and 88.00 MPa, 39.90 MPa and 89.05 MPa, and 38.90 MPa and 86.90 MPa, respectively.

The control mix showed high strength, as did some of the other mixes. Reductions in the strengths of some mixed specimens were attributed to the porous structure of the used materials and delayed hydration. Even though no fibers, SCMs, or other fillers were used to enhance the strength of the specimens, the strengths approached 40.00 MPa and 80.00 MPa with a low w/c of 0.2 after demolding and curing in water for three days, respectively.

### 3.6. Flexural Strength

**Figure 9:**
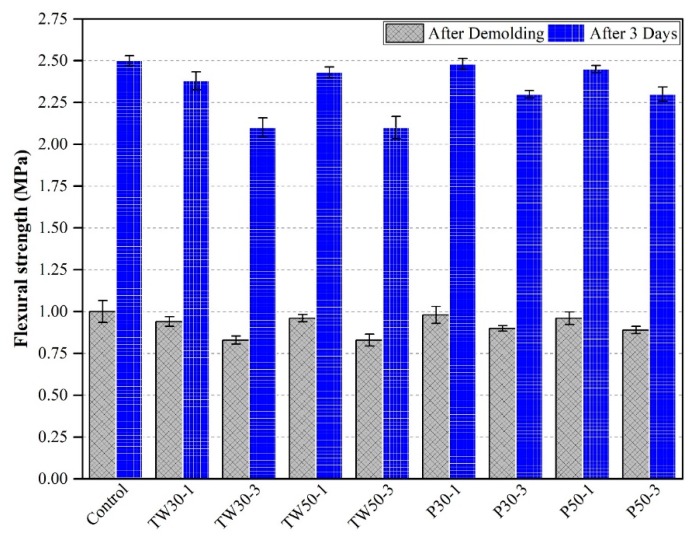
Flexural strength of specimens.

The flexural strengths of all prismatic mortar specimens after demolding and curing in water for three days are shown in Figure 9. The control mix had flexural strengths of 1.0 MPa and 2.5 MPa after demolding and curing in water for three days, respectively. The flexural strengths of TW30-1, TW30-3, TW50-1, TW50-3, P30-1, P30-3, P50-1, and P50-3 after demolding and curing in water for three days were 0.94 MPa and 2.38 MPa, 0.83 MPa and 2.10 MPa, 0.96 MPa and 2.43 MPa, 0.83 MPa and 2.10 MPa, 0.98 MPa and 2.48 MPa, 0.90 MPa and 2.30 MPa, 0.96 MPa and 2.45 MPa, and 0.89 MPa and 2.30 MPa, respectively.

The obtained flexural strength test results from this work were consistent with the literature. The control mix was stronger than all other eight mixes. The reduction in flexural strength of the mixed specimens was attributed to the porous structure of the materials used (tea waste and perlite) and delayed hydration. 

### 3.7. Ultrasonic Pulse Velocities

**Figure 10:**
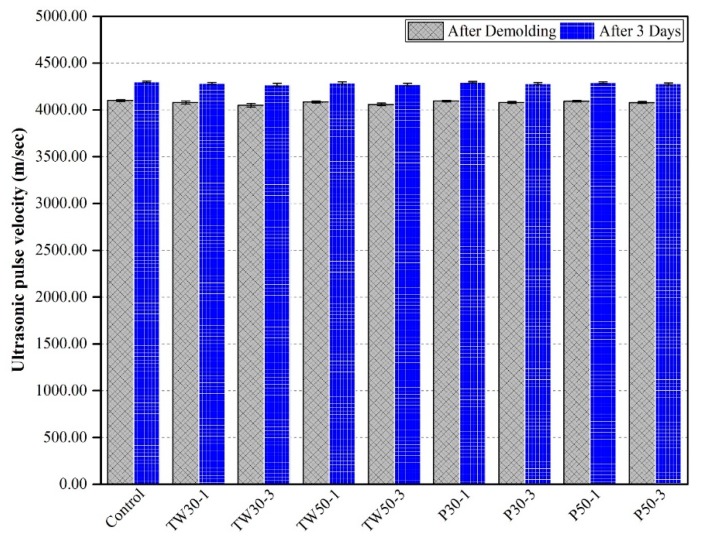
Ultrasonic pulse velocities of specimens.

Ultrasonic pulse velocity (UPV) results for all prismatic mortar specimens after demolding and curing in water for three days are shown in Figure 10. The control mix had velocities of 3600 m/s and 4300 m/s after demolding and curing, respectively. The remaining eight mixes had slightly lower velocity values than the control mix. The UPV of the remaining eight mixes (TW30-1, TW30-3, TW50-1, TW50-3, P30-1, P30-3, P50-1, and P50-3) were 3560 m/s and 4285 m/s, 3500 m/s and 4270 m/s, 3574 m/s and 4289 m/s, 3520 m/s and 4275 m/s, 3591 m/s and 4291 m/s, 3550 m/s and 4280 m/s, 3596 m/s and 4293 m/s, and 3555 m/s and 4281 m/s, respectively. The addition of tea waste and perlite particles reduced the velocities of the specimens. This reduction might be due to the presence of porous ICAs. The effect of porosity may vanish over time when complete hydration occurs and a denser microstructure forms.

The compressive strength, flexural strength, and UPV results from this research work were also consistent with what has been reported previously in the literature. Song et al. [34] found a reduction in strength as the dosage of SAPs was increased. However, the strength increase ratio from 7–28 days was observed to be larger in the specimens that contained SAPs than those that did not. Here, the later age strength at 28 days was attributed to the internal curing effect of SAPs. Craeye et al. [41] found that the addition of internal curing agents had an adverse effect on the mechanical properties of concrete because the strength of the specimens was found to be reduced. Geiker et al. [13] added 0.4% SAP by weight of cement in mortars at a 0.35 w/c ratio. They found 20% promotion of compressive strength as compared to the control mix after 28 days. The gain in strength was attributed to long-term hydration due to the presence of SAPs. However, Pierard et al. [42] used 0.3–0.6% SAPs by weight of total used powders and observed a reduction in strength after 28 days. A literature survey by [22] concluded that the addition of perlite decreased the strength. 

## 4. Conclusions

The basic purpose of this study was to use tea waste and perlite particles to minimize the inner heat release rate and delay hydration process of cement-based mortars. Moreover, we achieved our desired set goals, and the use of tea waste was proven to be a possible internal curing agent to delay early age hydration. The detailed conclusion remarks are given below:

- Delay in early age hydration and reduction in leading interior temperature was observed in the mixes that contained tea waste and perlite particles. More delay in hydration and reduction in leading temperature was observed in mixes that contained coarser particles of tea waste and perlite with the addition up to 3% by volume of cement.
- Mixed specimens that contained saturated tea waste and perlite particles had extended setting times. Specimens that contained 3% coarse tea waste and perlite particles had longer setting times than the other mixes. Moreover, the setting times of mixes that contained tea waste particles had higher setting times than specimens made with perlite particles.
- The density and weight of the control mix (OPC) was higher than that of the other mixes. Specimens that contained coarser tea waste and perlite particles (3% addition) exhibited reduced densities and weights, as compared to specimens made with finer particles. This was attributed to lower densities of the materials used and the compaction differences.
- High absorption values were obtained in the mix specimens that contained up to 3% coarser tea waste and perlite particles. The absorption rate of the control mix (OPC) was lower than that of all other mixes. The high absorption rate was attributed to the porous nature of the tea waste and perlite particles used.
- The compressive and flexural strengths of the control mix (OPC) were higher than those mixes that contained tea waste and perlite particles. The reduction in strength in mixes that contained internal curing agents (tea waste and perlite particles) was attributed to the porous nature of specimens.
- The ultrasonic pulse velocity (UPV) of the control mix (OPC) was higher than those of mixes that contained tea waste and perlite particles. Similar to the strength properties, it was attributed to the porous nature of specimens.

## 5. Future Work under Consideration

We plan to further explore the utilization of tea waste as an internal curing agent in high-strength cement composites to mitigate early age shrinkage (chemical and autogenous shrinkage) and to determine the effect of tea waste on concrete strength, microstructure, and durability against chloride penetration, sulfate attack, freeze and thaw, and carbonation for long periods of time.

## Figures and Tables

**Table 1:** Physical properties of sand, tea waste, and perlite particles. OPC, ordinary Portland cement; TW, tea waste; P, perlite.

Description	Results					
Physical Properties	OPC	Dry Sand	Dry TW30	Dry TW50	Dry P30	Dry P50
Specific Gravity (g/cm^3^)	3.15	2.65	0.342	0.465	0.345	0.650
Absorption Ability (%)	-	4.5	215	110	75.0	47.0
Moisture Content (%)	-	0.2	3.5	2.1	2.1	1.5
Blaine Fineness (m^2^/kg)	325	-	-	-	-	-

**Table 2:** Mix proportions of mortar specimens.

Mix ID	OPC by Weight	Cement/Sand by Weight	Water/Cement by Weight	TW30% by Volume of Cement	TW50% by Volume of Cement	P30% by Volume of Cement	P50% by Volume of Cement	S.P/Cement by Weight
Control	1.0	1.25	0.2	-	-	-	-	0.01
TW30-1				1.0	-	-	-	
TW30-3				3.0	-	-	-	
TW50-1				-	1.0	-	-	
TW50-3				-	3.0	-	-	
P30-1				-	-	1.0	-	
P30-3				-	-	3.0	-	
P50-1				-	-	-	1.0	
P50-3				-	-	-	3.0	
